# Study on Autolytic Mechanism and Self-Healing Properties of Autolytic Clinker Microsphere in Alkaline Environment

**DOI:** 10.3390/ma15103638

**Published:** 2022-05-19

**Authors:** Jun Li, Wenting Li, Zhengwu Jiang

**Affiliations:** Key Laboratory of Advanced Civil Engineering Materials of Ministry of Education, School of Materials Science and Engineering, Tongji University, Shanghai 201804, China; 1610421@tongji.edu.cn (J.L.); lwt@tongji.edu.cn (W.L.)

**Keywords:** mineral self-healing, alkaline environment, autolytic clinker microsphere, autolytic mechanism

## Abstract

In this study, the autolytic clinker microsphere with clinker as core and polyvinyl pyrrolidone (PVP) as coating film was prepared. Pretreatment of clinker with silane coupling agent was firstly processed during the preparation. To investigate the autolytic mechanism, the microstructures of the autolytic clinker microsphere at different curing ages were observed using environmental scanning electron microscopy (ESEM), equipped with an energy dispersive spectrometer (EDS). The autolytic stages were also identified based on the microstructural evolution. The influence of pretreatment degree on autolytic behavior was also studied by measurements of micro-morphology and isothermal calorimetry. Experimental results indicated that the compressive strength recovery of specimens was increased by 15–19% due to the addition of autolytic clinker microspheres. The recovery of compressive strength was also improved with the increase of pH value. The improvements in compressive strength recovery of specimens with microspheres were in the range of 15–19%, 15–31%, 25–36%, and 29–50% with the pH value of 7, 8, 10, and 12, respectively. It was also found that inner damage of cement-based matrix had greater recovery when pre-cracked specimens were cured in alkaline environments.

## 1. Introduction

Concrete is the most common building structure material in the modern world, which has been widely used in construction, tunnel, bridge, and other concrete structure engineering. The wide application of concrete is owing to its high mechanical properties, well molding performance, low production cost, and simple production process [1,2]. Although concrete has several practical advantages, concrete structures still face many problems, especially crack formation. The cracks of concrete are the main factor affecting structural integrity and serviceability [3,4]. Cracks are inevitable due to the brittleness of concrete. Once the cracks are connected, the deterioration of concrete will be accelerated [5,6,7]. There are many research works focusing on improving the mechanical properties and durability of concrete [8,9,10]. Meanwhile, conventional repair and maintenance are usually adopted to extend the service life of the concrete structure. However, the cost of manual maintenance can be prohibitively expensive, especially for large infrastructure. Moreover, it is difficult to repair where works are in continuous service structures or underground concrete structures such as highways and tunnels, or even impossible to carry out repair work where cracks are not visible [11,12]. Therefore, crack self-healing of concrete is a more feasible and effective method, and many researchers pay attention to optimizing or innovating these self-healing methods.

The autogenous self-healing of cracks is a natural phenomenon in cement matrix, first noticed in 1836 [1]. The plain concrete possesses the ability to heal small cracks without any external intervention, because of the continuous hydration of unhydrated cement as well as carbonation of Ca(OH)_2_. However, the autogenous healing efficiency is limited and difficult to control [13,14]. Therefore, some external additions such as polymeric adhesive agents, bacteria, and mineral admixtures are incorporated into concrete to enhance the self-healing properties [15,16,17].

Recent research makes the classification of self-healing methods based on whether there are any host-guest chemical interactions [18]. Intrinsic healing methods present better healing effectiveness due to the chemical interaction with cement-based matrix. Mineral self-healing, as one of the intrinsic healing, is proposed by many researchers due to the obtained healing products presenting better compatibility [19,20]. Also, the addition of minerals usually does not reduce the strength of the concrete matrix too much [21]. Clinker, as one of the original concrete components, is also adopted as a potential mineral to enhance the self-healing properties of cement-based materials [22]. However, the direct addition of adopted mineral admixture may reduce the self-healing potential due to premature consumption during mixing. Therefore, encapsulation technology is applied to solve this problem [23,24,25,26]. The well-encapsulated mineral could better increase the compressive and flexural strength [27].

An autolytic microsphere was proposed in previous researches, and the self-healing potential is preliminarily confirmed [28,29]. This novel mineral self-healing method proposes a core/shell autolytic microsphere. The core healing mineral admixture includes expansive admixture, geomaterials, or crystalline admixtures. Polyvinyl pyrrolidone (PVP) is adopted as the shell material. PVP film has high tensile strength and can withstand shear forces, so that the film will not break in the mixing process [30,31]. The PVP film is designed to avoid premature consumption of mineral admixtures, which is gradually autolysis in hardened paste due to an alkaline solution. The core mineral is ready for crack healing and remains dormant until cracks occur and water penetrates [28]. Therefore, researches on the autolytic process of this microsphere are very important [32].

In this study, the autolytic clinker microsphere was chosen as a self-healing microsphere. This autolytic clinker microsphere was prepared with clinker as a healing mineral, PVP as coating film and silane coupling agent as a pretreated agent of clinker. To further study the autolytic process, the microstructures of the autolytic clinker microsphere at different curing ages were observed to study the autolytic mechanism. The effect of pretreatment degree on autolytic behavior was also determined. In addition, the influence of alkaline environments on self-healing properties was evaluated by measurement of compressive strength and ultrasonic pulse time to investigate the environmental factors influencing the self-healing properties.

## 2. Materials and Methods

### 2.1. Raw Materials

The used autolytic clinker microsphere was mainly composed of clinker and PVP ((C_6_H_9_NO)_n_, molecular weight is 40,000). The clinker was chosen as the potential healing mineral. To avoid the premature consumption of clinker, PVP was selected as the coating film material. In addition, surface pretreatment of cement clinker with silane coupling agent (C_6_H_17_NO_3_S, γ-aminopropyl triethoxysilane) was taken to provide an active binding layer, which could make the coating film more effective. The above chemical agents (PVP and silane coupling agent) in the analytic grade were produced by Sinopharm Chemical Reagent Co., Ltd. (Shanghai, China). 

**Figure 1:**
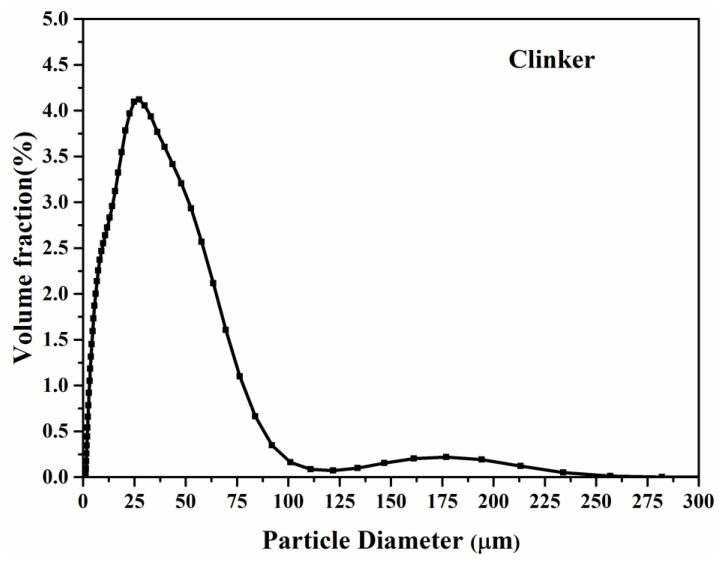
Particle size distribution of used clinker.

The particle size distribution and chemical compositions of the used clinker with a density of 3.16 g/cm^3^ are shown in Figure 1 and Table 1, respectively. The chemical compositions of Portland cement with a density of 3.14 g/cm^3^ used for specimen preparation are also listed in Table 2. The X-ray fluorescence (XRF) analysis based on SRS 3400 spectrometer (Bruker, Borken, Germany) was used to measure the chemical compositions of clinker and Portland cement. The tableting samples of clinker and cement powders were used for XRF analysis.

### 2.2. Preparation of Autolytic Clinker Microsphere

**Figure 2:**
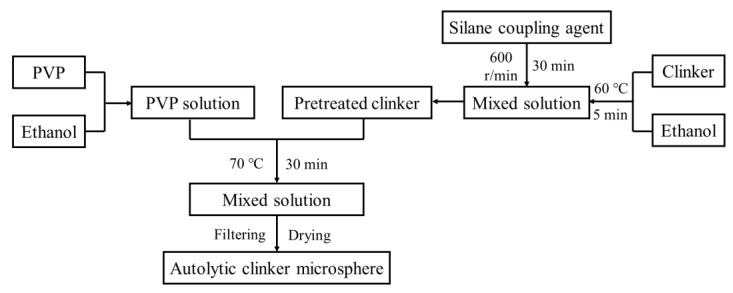
Schematic illustration of autolytic clinker microsphere preparation process.

The preparation of the autolytic clinker microsphere involved two steps. The first step was the pretreatment of clinker. The clinker of 100 g and silane coupling agent of 8 g (different amounts of silane coupling agent of 1 g, 3 g, 5 g, and 8 g only for autolytic behavior test) were stirred in an ethanol solution of 100 mL, and then filtering and drying were taken to obtain pretreated clinker. The second step was film coating of the pretreated clinker. The PVP of 5 g and pretreated clinker of 100 g were stirred in an ethanol solution of 300 mL, and the final autolytic clinker microsphere was also obtained through filtering and drying. In this experiment, a constant mass ratio of PVP to clinker equaling to 1/20 (film thickness with 5.75 μm) was adopted to study the autolytic mechanism and self-healing properties of the prepared autolytic clinker microsphere. The pretreated clinker and autolytic clinker microsphere were collected by washing three times with ethanol solution and then dried at 105 °C. The used stirring speed and heating temperature ensured that the reaction of clinker, silane coupling agent, and PVP fully proceeded. The detailed preparation process was already introduced in previous research [29], and illustrated in Figure 2.

### 2.3. Mixture Proportion and Specimen Preparation

In this study, cement paste specimens were prepared with 0.4 w/c. The dimensions of 25 × 25 × 25 mm for compression test and 40 × 40 × 160 mm for damage degree test were prepared with manual vibration, respectively. The paste mixing method was performed according to Chinese standards (GBT 1346-2011). The hardened paste was immediately cured in the curing room (with a temperature of 20 ± 2 °C and RH ≥ 95%) after demolding. After subsequent curing for 27 days, the 80% compression load of the average ultimate force was adopted to pre-crack the specimens for the compressive test, resulting in internally distributed microcracks [7]. Meanwhile, the specimens for the damage degree test were preloaded at 60% of maximum flexural force by the four-point bending test. Then both pre-cracked specimens were cured in different alkaline environments until the following test day. This experiment mainly studied the effects of the different alkaline environments on the self-healing properties, so a constant autolytic clinker microsphere replacement rate of 20% by cement mass was adopted.

### 2.4. Test Methods

#### 2.4.1. Morphology of Autolytic Clinker Microsphere

The microstructures of the autolytic clinker microsphere were observed at different curing ages. The cement paste specimen was broken and the central sample was taken for backscattered electron imaging (BSE) observation. Environmental scanning electron microscopy (ESEM, Quanta 200F) (FEI company, Hillsboro, OR, USA) was used to investigate the microstructure, equipped with an energy dispersive spectrometer (EDS) (FEI company, Hillsboro, OR, USA). 

The clinker and pretreated clinker with silane coupling agent of 8 g were collected immediately after preparation, and were coated with gold before the observation of secondary electrons images.

The prepared cement specimens with autolytic clinker microsphere were placed in a curing room (20 ± 2 °C and RH ≥ 95%) for 1 day before demolding. Then the specimens were completely immersed in water for 4 h, 24 h, and 48 h to perform BSE tests. The central parts of paste specimens were taken and sealed with epoxy resin, and then the sample surface was polished carefully. Before BSE observation, the surface was coated with carbon.

#### 2.4.2. Autolytic Behavior

The autolytic behavior of the microsphere is described by autolytic time, which is defined as the required time for complete film autolysis [28,29]. Therefore, the heat flow of autolytic clinker microsphere paste was determined by isothermal calorimetry according to Chinese standards (GB12959-2008). The measuring system of semi-adiabatic calorimeters was used [33]. The autolytic clinker microsphere paste contained the prepared autolytic clinker microsphere and water, where the mass ratio of water to microsphere was 0.4. The autolytic time is calculated according to Equation (1).

T = Tm − Tc,(1)

where T is the calculated autolytic time (min). Tm is the time when the hydration exothermic peak of autolytic clinker microsphere paste occurs (min). Tc is the time when the hydration exothermic peak of reference cement paste occurs (min).

#### 2.4.3. Self-Healing Properties

In this experiment, the self-healing properties were determined by recovery of compressive strength and damage degree. Since Ca^2+^ is important to healing effectiveness, the Ca(OH)_2_ solutions with different initial pH levels, measured by pH meter (PHS-25) (INE SA Scientific Instrument Company, Shanghai, China), were utilized to simulate the alkaline environments. Considering the possible crystallization of saturated calcium oxide solution with a pH of 12.6 at 20 °C, Ca(OH)_2_ solutions with initial pH of 7, 8, 10, and 12 were adopted.

The compressive strength of pre-cracked specimens was tested after alkaline curing age of 2 days, 14 days, and 28 days. Three replicates were measured in the same test. The compressive strength recovery is determined according to Equation (2).

η = Pr/Pi,(2)

where η calculates the recovery of compressive strength (%). Pr is the average ultimate compressive strength after self-healing (MPa). Pi is the initial compressive strength in the pre-cracking test (MPa).

The recovery of damage degree was determined by ultrasonic pulse test after alkaline curing age of 2 days, 7 days, 14 days, and 28 days. The change of inner damage was monitored by one specimen. The direction of pulse transmission was vertical to the loading force direction. The damage degree is calculated according to Equation (3)

Dm = 1 − (T_b_/T_a_)^2^, (3)

where Dm is the calculated damage degree. T_b_ is the transmitting time before preloading. T_a_ is the transmitting time after self-healing at specific curing age.

## 3. Results and Discussion

### 3.1. Autolytic Mechanism

#### 3.1.1. Autolytic Stage of Microsphere

**Figure 3:**
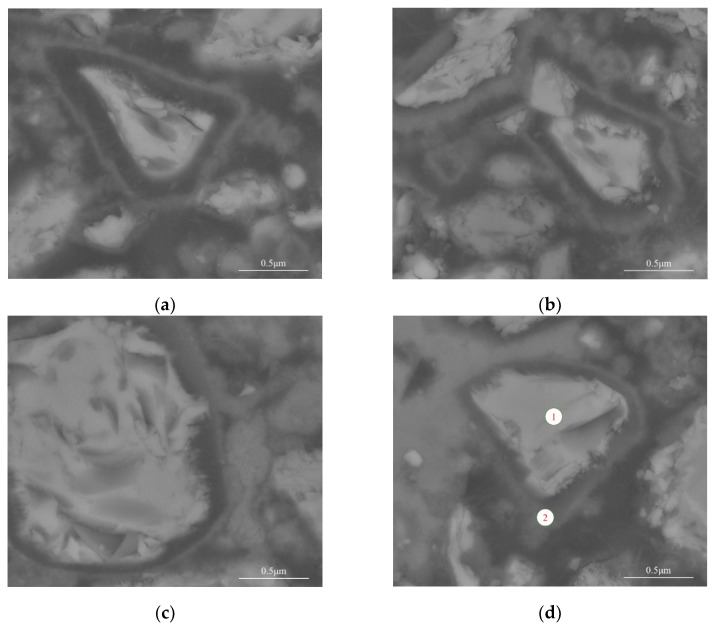
Microstructure evolution of autolytic clinker microspheres at (**a**) curing room for 1 day, (**b**) curing room for 1 day and then water curing for 4 h, (**c**) curing room for 1 day and then water curing for 24 h, (**d**) curing room for 1 day and then water curing for 48 h.

The microstructures of the autolytic clinker microsphere at different curing ages were observed. The results are shown in Figure 3.

Results from Figure 3a show that the autolytic clinker microsphere remains coating film after mixing. The dark regions in the figures are PVP film while the brightest regions in the middle are encapsulated mineral that is clinker according to previous EDS analysis [29]. As the hydration proceeded, the thickness and integrity of the coating film changed. Partial autolysis of the coating film occurred, shown in Figure 3b. Moreover, coating film has more autolytic behavior and becomes thinner, easily seen in Figure 3c. Meanwhile, it is can be seen from Figure 3d that the coating film of the autolytic clinker microsphere finally disappears when the specimen is cured for a total of 72 h. It is inferred that with the thinning of the coating film, the thickness of the hydration layer partially attached to the outer surface of the clinker gradually increases. The results of EDS spot analysis on regions of Figure 3d are shown in Figure 3 and Table 3 to further study the autolytic mechanism.

**Figure 4:**
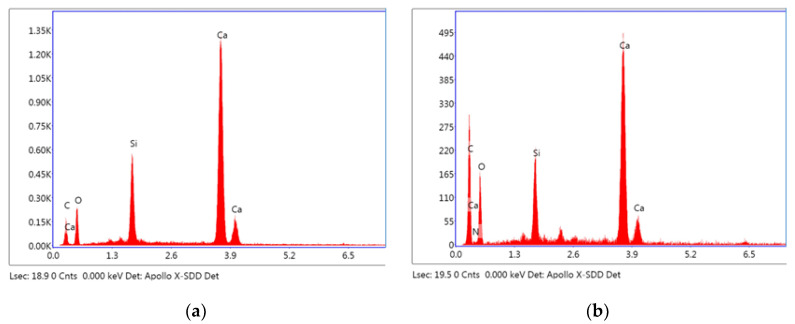
The EDS spot analysis of autolytic clinker microspheres (**a**) for point 1, (**b**) for point 2.

The results from Figure 4 and Table 3 show that the main elements of point 1 are Ca and O, accounting for 80.74% of the total elements by weight. The brightest regions are clinkers with mass ratio of calcium to silicon equaling 4.43 [29,34]. The mass percentage of the Ca element decreases significantly in point 2 with the mass ratio of Ca to Si equaling 5.19, which is probably due to the early consumption of C_3_S and C_3_A. From Figure 4b, it is also noticed that there is a small peak of the N element, which confirms the existence of PVP. Meanwhile, the mass percentage of the C element increases at point 2. Therefore, it is inferred that the clinker is surrounded by hydration products, epoxy resin (probably microcracks and leaved regions of PVP film after autolysis) and a small amount of PVP. These results show that the coating film can protect the inner healing mineral substance well in the early hydration. The autolysis of coating film occurs with hydration proceeding and some new hydration products will partially fill the leaved regions of the coating film after autolysis.

Based on the above results, the autolytic mechanism of microspheres is explored and the autolytic stage is divided into three processes. (1) Autolytic clinker microspheres remain almost intact. In this process, remained coating film avoids premature consumption during mixing. (2) The coating film becomes thinner. In this process, the autolysis of coating film occurs gradually, which also protects the clinker from reaction with water. (3) Complete autolysis of coating film. In this process, most PVP films are autolysis. Some new hydration products partially fill the leaved regions of the coating film after autolysis.

#### 3.1.2. Effect of Pretreatment Degree on Autolytic Behavior

**Figure 5:**
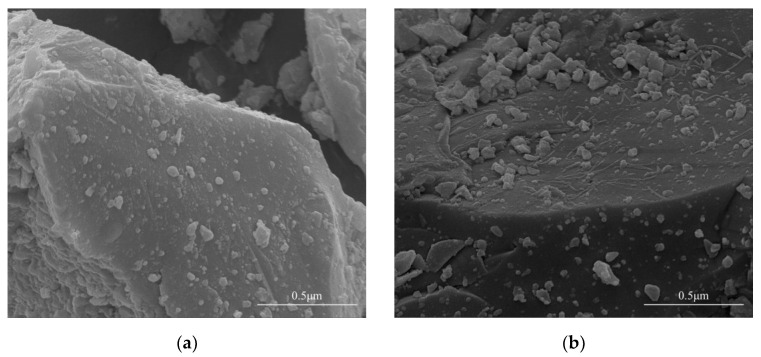
The microstructures of (**a**) clinker without pretreatment and (**b**) pretreated clinker.

In the preparation procedure of autolytic clinker microspheres, pretreatment of clinker should be performed firstly, and the microstructures of clinker and pretreated clinker are illustrated in Figure 5.

Results show that there are many obvious flocculent-like attachments in Figure 5b, compared with the microstructure of clinker without pretreatment in Figure 5a. This indicates that silane coupling agents are successfully connected on the surface of clinker, providing an active binding layer [29]. To study the influence of pretreatment degree on autolytic behavior, the heat flow of pretreated clinker paste and autolytic clinker microsphere paste with different addition of silane coupling agent were determined, as shown in Figure 5.

The pretreated clinker pastes with silane coupling agent of 1%, 3%, 5%, and 8% by cement weight are noted as 1%SCA, 3%SCA, 5%SCA, 8%SCA, respectively. The autolytic clinker microsphere pastes with silane coupling agents of 1%, 3%, 5%, and 8% by cement weight are noted as K-1, K-3, K-5, and K-8, respectively. K-0 is the original cement paste compared with cement clinker paste to confirm the feasibility of measurements.

**Figure 6:**
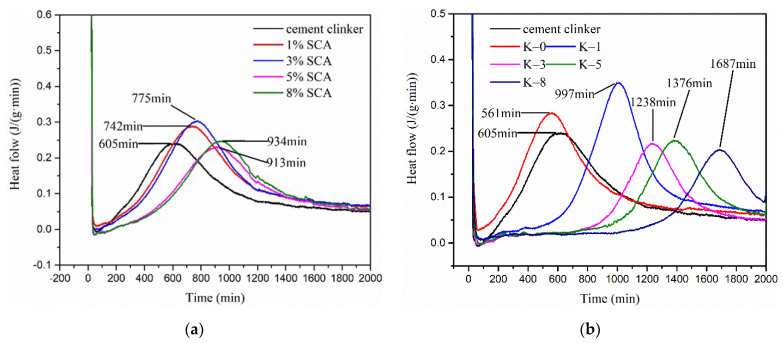
Different additions of silane coupling agent on the heat flow of (**a**) pretreated clinker paste without PVP film and (**b**) autolytic clinker microsphere paste with PVP film.

Results in Figure 6a show that the hydration exothermic peaks are delayed with the increase of the silane coupling agent from 1% to 8%. The autolytic times are calculated as 137 min, 170 min, 308 min, and 329 min, respectively. This also indicates that the clinker is successfully pretreated with a silane coupling agent. The limited improvements of clinker pastes are achieved in terms of autolytic time. Figure 6b shows the effect of pretreatment degree on the heat flow of autolytic clinker microsphere pastes. As the addition of silane coupling agent increases from 1% to 8%, the autolytic times are calculated as 392 min, 633 min, 771 min, and 1082 min, respectively. The autolytic time has been greatly improved due to the PVP coating film. In the case of the same pretreatment degree from 1% to 8%, the autolytic times of autolytic clinker microsphere pastes increase by 186%, 272%, 150%, and 229% compared with pretreated clinker paste. This indicates that pretreatment of clinker greatly enhances the autolytic time of autolytic clinker microsphere pastes. The pretreatment process is very necessary for the autolytic clinker microsphere preparation. Experimental results show that the pretreatment of clinker is effective and presents significant improvements in terms of autolytic time.

### 3.2. Recovery of Compressive Strength in Different Alkaline Environments

The recovery of compressive strength in different alkaline environments was determined. The tested specimens were categorized into two groups. The reference group of pre-cracked specimens were cured in water, noted as pH = 7 without microspheres. The experimental group of pre-cracked specimens were cured in different alkaline environment, noted as pH = 7, pH = 8, pH = 10 and pH = 12. Ca(OH)_2_ solutions with different initial pH levels were adopted to simulate the alkaline environment.

**Figure 7:**
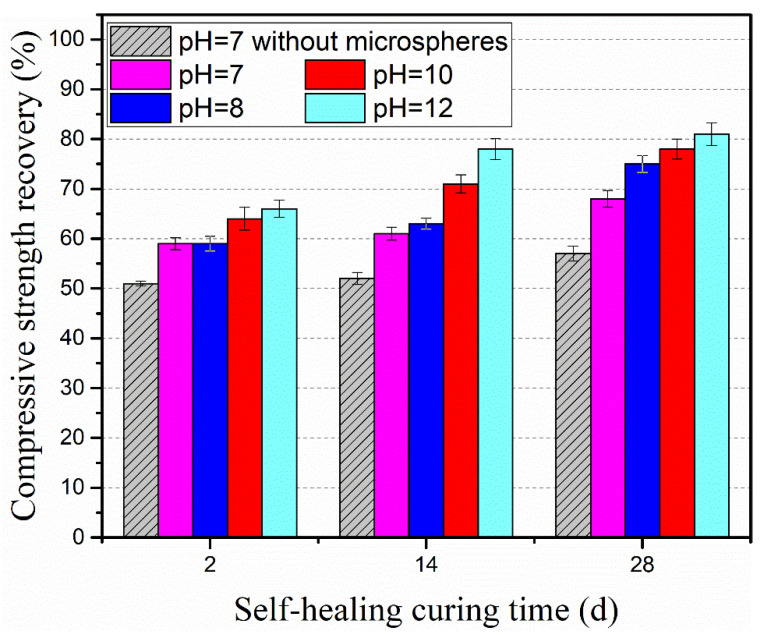
Recovery of compressive strength in different alkaline environments.

Results from Figure 7 show that all specimens with autolytic clinker microspheres present a much higher recovery of compressive strength than specimens without autolytic clinker microspheres at the same curing age. In the curing condition of pH = 7, the compressive strength recovery of specimens with the addition of autolytic clinker microspheres increases by 15%, 17%, and 19% at the curing age of 2 days, 14 days, and 28 days, respectively. This indicates that the addition of autolytic clinker microspheres will improve the compressive strength recovery. In the curing conditions of the different alkaline environments, compared with reference specimens, the improvements in compressive strength recovery of specimens with microspheres are in the range of 15–19%, 15–31%, 25–36%, and 29–50% with the pH value of 7, 8, 10, and 12, respectively. Results show that the recovery of compressive strength gets improved with the increase of pH value. Meanwhile, it is found that there is a limited improvement at the curing age of 2 days with compressive strength recovery of 59–66%. The better improvements are obtained at the curing age of 14 days with compressive strength recovery of 61–78%, and at the curing age of 28 days with compressive strength recovery of 68–81%. It is inferred that the autolytic clinker microspheres provide more clinker for cement-based matrix ready to heal microcracks, and the clinker is more beneficial to improve the compressive strength recovery at later curing age. The alkaline environment will improve the compressive strength recovery probably due to the lower porosity [35].

### 3.3. Damage Degree in Different Alkaline Environments

**Figure 8:**
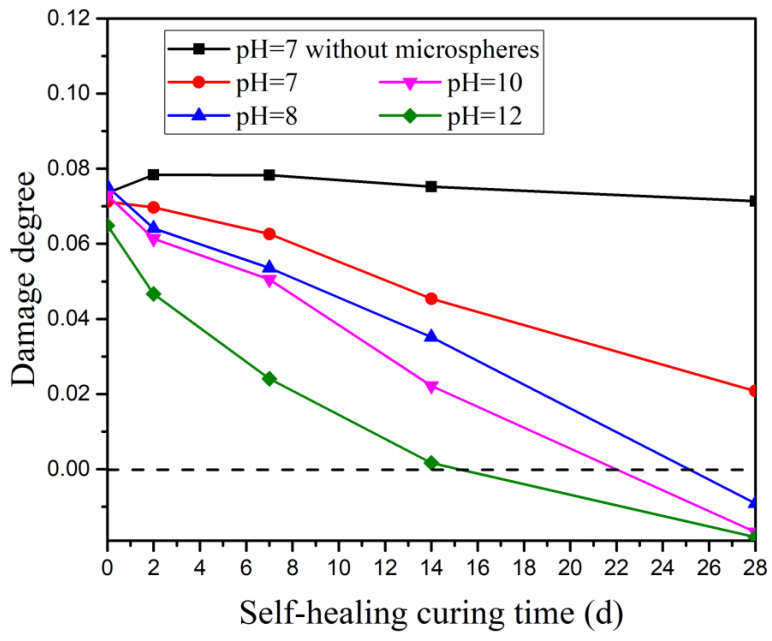
Influence of different alkaline environments on damage degree.

Determination of damage degree is an effective and non-destructive measurement to determine the information about inner porous structures and microcracks of concrete based on the ultrasonic pulse test [36]. The decrease in damage degree reflects the recovery of inner damage in concrete [37]. The variation of the alkaline environment influencing damage degree is illustrated in Figure 8. The notations represent the specimens cured in the same alkaline environment as the test of compressive strength recovery.

Results from Figure 8 show a similar influencing trend as the test of compressive strength recovery. The damage degree of the reference specimen (without autolytic clinker microspheres) presents a small reduction. It is inferred that the inner damage of the reference specimen gets limited recovery. However, the experimental specimens (with autolytic clinker microspheres) show much more reduction in terms of damage degree. In the curing condition of pH = 7, compared with the reference specimen, the damage degree of the experimental specimen decreases by 3%, 11%, 20%, 39%, and 70% at the curing age of 2 days, 7 days, 14 days, and 28 days, respectively. This indicates that the addition of an autolytic clinker microsphere is beneficial to recovering the inner damage. In the curing condition of the different alkaline environments, the damage degree decreases with increasing the pH value. It is inferred that inner damage of cement-based matrix gets more recovery when pre-cracked specimens are cured in alkaline environments. Meanwhile, in the curing condition of pH greater than 7, the damage degrees of experimental specimens fall below the zero-standard line at the curing age of 28 days, which means further recovery of inner damage besides the damage induced by bending force [38]. Experimental results show that the alkaline environment improves inner damage more effectively than compressive strength recovery.

## 4. Conclusions

In this paper, an autolytic clinker microsphere was prepared and research on the autolytic mechanism was carried out. The autolytic stages with hydration proceeding are determined. The influence of an alkaline environment on self-healing properties was also evaluated. The following conclusions are obtained from the experimental research.

1. The autolytic mechanism of autolytic clinker microspheres is determined by tracing the microstructure evolution with hydration proceeding. Three autolytic stages are identified;
2. Pretreatment of clinker is confirmed effectively through measurements of micro-morphology and heat flow. The increasing amount of silane coupling agent and addition of PVP film will increase the autolytic time;
3. The compressive strength recovery and damage degree are improved with the addition of autolytic clinker microspheres and also improved by curing in alkaline environments.
4. The alkaline environment improves inner damage more effectively than compressive strength recovery.

Further research is needed to study the autolytic mechanism of the autolytic clinker microsphere. The evolution of the chemical composition of silane coupling agents and PVP film is worth investigating. In addition, more self-healing properties should be determined such as crack width and water permeability.

## Figures and Tables

**Table 1:** Chemical compositions of used clinker.

Component	SiO_2_	CaO	Fe_2_O_3_	Al_2_O_3_	MgO	SO_3_	Na_2_O	K_2_O	Other
Content (wt.%)	24.1	63.1	3.27	4.34	1.16	0.76	0.12	0.8	2.35

**Table 2:** Chemical compositions of used Portland cement.

Component	SiO_2_	CaO	Fe_2_O_3_	Al_2_O_3_	MgO	TiO_2_	SO_3_	Na_2_O	K_2_O	LOI
Content (wt.%)	20.0	64.3	3.11	4.51	0.68	0.22	2.99	0.03	0.72	2.70

**Table 3:** Mass distribution of major elements in different points from Figure 3d.

Element Weight %	Ca	Si	C	O
Point 1	41.47	9.36	9.90	39.27
Point 2	23.06	4.44	34.29	38.13

## Data Availability

Data sharing is not applicable to this article.
